# Metabolic model of necrotizing enterocolitis in the premature newborn gut resulting from enteric dysbiosis

**DOI:** 10.3389/fped.2022.893059

**Published:** 2022-08-23

**Authors:** Giorgio Casaburi, Jingjing Wei, Sufyan Kazi, Junlin Liu, Kewei Wang, Guo-Zhong Tao, Po-Yu Lin, James C. Y. Dunn, Bethany M. Henrick, Steven A. Frese, Karl G. Sylvester

**Affiliations:** ^1^Evolve Biosystems, Inc., Davis, CA, United States; ^2^Department of Surgery, Stanford University, Stanford, CA, United States; ^3^Department of Pediatrics, Shanxi Medical University, Taiyuan, China; ^4^Department of General Surgery, The People's Hospital of Liuyang City, Liuyang, China; ^5^Department of Gastrointestinal Surgery, The First Hospital of China Medical University, Shenyang, China; ^6^Department of Food Science and Technology, University of Nebraska, Lincoln, NE, United States; ^7^Department of Nutrition, University of Nevada Reno, Reno, NV, United States

**Keywords:** necrotizing enterocolitis (NEC), microbiome, formate, metagenomics, artificial intelligence, dysbiosis, probiotics

## Abstract

Necrotizing enterocolitis (NEC) is a leading cause of premature newborn morbidity and mortality. The clinical features of NEC consistently include prematurity, gut dysbiosis and enteral inflammation, yet the pathogenesis remains obscure. Herein we combine metagenomics and targeted metabolomics, with functional *in vivo* and *in vitro* assessment, to define a novel molecular mechanism of NEC. One thousand six hundred and forty seven publicly available metagenomics datasets were analyzed (NEC = 245; healthy = 1,402) using artificial intelligence methodologies. Targeted metabolomic profiling was used to quantify the concentration of specified fecal metabolites at NEC onset (*n* = 8), during recovery (*n* = 6), and in age matched controls (*n* = 10). Toxicity assays of discovered metabolites were performed *in vivo* in mice and *in vitro* using human intestinal epithelial cells. Metagenomic and targeted metabolomic analyses revealed significant differences in pyruvate fermentation pathways and associated intermediates. Notably, the short chain fatty acid formate was elevated in the stool of NEC patients at disease onset (*P* = 0.005) dissipated during recovery (*P* = 0.02) and positively correlated with degree of intestinal injury (*r*^2^ = 0.86). *In vitro*, formate caused enterocyte cytotoxicity in human cells through necroptosis (*P* < 0.01). *In vivo*, luminal formate caused significant dose and development dependent NEC-like injury in newborn mice. *Enterobacter cloacae* and *Klebsiella pneumoniae* were the most discriminatory taxa related to NEC dysbiosis and increased formate production. Together, these data suggest a novel biochemical mechanism of NEC through the microbial production of formate. Clinical efforts to prevent NEC should focus on reducing the functional consequences of newborn gut dysbiosis associated metabolic pathways.

## Importance

Necrotizing enterocolitis (NEC) is a leading cause of premature newborn morbidity and mortality, and correlated with enteric dysbiosis and inflammation; however, the pathogenesis remains obscure. Here, we utilized publicly available metagenomic sequences and proprietary targeted metabolomics combined with functional *in vivo* and *in vitro* assessment, to define a novel molecular mechanism of NEC. Importantly, our analyses revealed a functional consequence of the metabolic capacity of newborn gut dysbiosis as a novel target for clinical efforts in premature infant care.

## One sentence summary

In newborns with NEC, enteric dysbiosis confers the metabolic capacity for enteric injury through altered fermentation patterns.

## Introduction

Necrotizing Enterocolitis (NEC) remains a leading cause of premature newborn morbidity and mortality (1–3). Despite decades of fundamental and clinical research, the precise pathogenesis of NEC remains elusive. NEC has been most durably associated with degree of prematurity, enteral feeding and intestinal dysbiosis favoring Proteobacteria predominant colonization, as well as the alteration in bacterial metabolites including short chain fatty acids (SCFA) (4–7). Furthermore, despite the well-documented presence of intestinal dysbiosis preceding NEC onset in longitudinal studies (5), a specific causal mechanism linked to distinct bacterial taxa has not been established. Dysbiosis in premature newborns leading to NEC has largely been characterized as absence of colonizing microbial heterogeneity, loss of specific taxa abundance and relative over-abundance of specific *Enterobacteraceae* species. Indeed, an abundance of newborn microbiome literature documents either altered microbiota diversity or aberrant bacterial taxonomy in association with specific disease states (8–11). It has become increasingly clear that gut dysbiosis is associated with various pathologies including NEC, but specific bacteria have not proven causative despite a variety of potential pathogenic features. Together, these observations represent an important gap in our understanding of the functional significance of premature newborn dysbiosis and suggest a still to be identified mechanism of pathophysiology conferred by colonizing microbes. Considering that most cases of NEC are observed in premature newborns that are being fed, one intriguing hypothesis that we sought to explore is that inciting pathologic features arise at the intersection of colonizing enteric microbial function and host features producing a biochemical pattern capable of producing enteric injury consistent with NEC.

Metagenomic sequencing has been increasingly used to better understand human colonizing microbiota and their functional potential in a variety of clinical settings, including hospitalized premature newborns (8, 12, 13). These approaches have revealed the effects of various perinatal exposures including antibiotics on the microbiota compositions and associated antibiotic resistance genes (14, 15). The absence of species level taxonomic classification and functional annotation of specific pathways and metabolites in association with NEC is an under-explored area of investigation. Thus, we recognized an opportunity to apply state-of-the-art sequencing and annotation techniques to functionally assess the microbiota at high resolution (gene and species level) in order to determine the degree to which microbe specific pathology may be biochemical in nature. Accordingly, in this study we sought to determine the relationship between NEC and the potential biochemical output of host-microbe-pathogenicity interactions as reflected in the metagenome, thus supporting a causal relationship between specific microbial bioproducts and NEC. Since the predominant product of microbiota fermentation of enterally provided substrates is organic acids, we further sought to determine if there was a significant shift in fecal stream short chain fatty acids (SCFA) associated with NEC that could precipitate enteric mucosal injury typical of NEC.

## Results

### Machine learning revealed NEC microbiome signatures by post-menstrual age

A total of 1,603 and 1,601 publicly available samples were selected after quality filtering for taxonomic and functional annotation, respectively (see methods). The largest dataset produced represented a matrix of 11,026,566 (UniRef90 hits) × 1,603 (samples; 245 NEC positive, 1,358 non-NEC) or 17.7 billion entries. Gene family entries were also converted into pathways. By default, HUMAnN2 uses MetaCyc pathway definitions and MinPath to identify a parsimonious set of pathways that explain observed reactions in the community. This led to a matrix of 1,601 (samples) × 595 (pathways) or 952.5 thousand entries. The microbial species identified in all samples were, in total, 4,786 (Supplementary Table 1).

For analysis, the sample size was divided into different subsets based on patient post menstrual age (PMA) at collection and machine learning techniques were applied to assess whether NEC or healthy preterm status could be predicted based on microbiome signatures.

First, models were trained at all taxonomic levels (phylum to species) and at several PMA combinations, which showed a moderate increase in sensitivity in models trained on samples with PMA >29 weeks (Supplementary Figures 1, 2). The model that performed the best was at the species level with samples >29 weeks PMA (testing accuracy = 0.9; precision = 0.24), a result that was similar to previously published NEC models (16).

**Figure 1:**
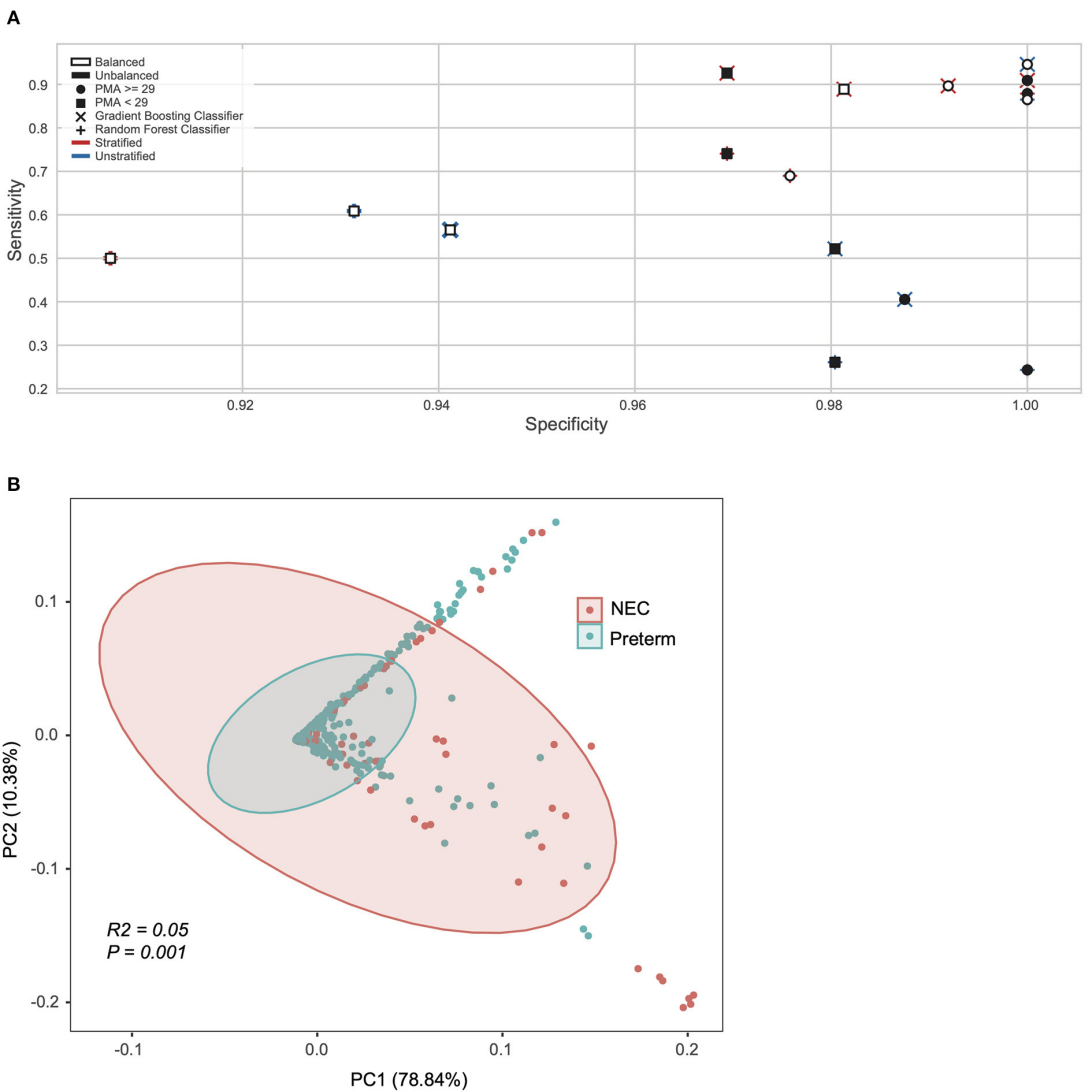
Machine learning revealed NEC microbiome signatures by post-menstrual age. **(A)** Pathway level data was stratified by species, oversampled to balance NEC classes, sub-setted by PMA, and trained on different model algorithms to explore model performance. Models sub-setted to include only samples from subjects with a PMA ≥29 weeks performed moderately better with respect to specificity and sensitivity than their counterparts. **(B)** Principal coordinate analysis (PCoA) of all samples based on weighted UniFrac distance and colored by Necrotizing Enterocolitis (NEC) or preterm infants without NEC (Preterm).

Next, we applied machine learning at the pathway level obtained from HUMAnN2 (see methods), testing pathway trends with or without the species stratification (e.g., a combination of all species to the same pathway or the same pathway considered as separate feature equal to the number of unique species contributing to that pathway). Sixteen different models were constructed on the significant pathways identified with the Gini-importance score varying between stratified vs. unstratified, balanced vs. unbalanced, and model architectures (Figure 1A). For these models the inputs were both stratified (*n* = 18,442 features) and unstratified (*n* = 597 features) microbial pathways and tested at both a PMA < 29 or ≥ 29 weeks, using balanced and unbalanced subsets for the minority class (NEC) and using random forest or gradient boosting classifiers (Figure 1A). The top performing models able to classify NEC at a higher specificity and sensitivity at the pathway levels, were all those with samples ≥29 PMA. Specifically, the best overall model was derived from a gradient booster classifier that had a combination of a balanced minority class, was taxonomically stratified, and had only ≥29 PMA (Figure 1A).

Based on the results obtained from the statistical analysis of taxonomy and pathways, we selected a subset of samples with a PMA ≥ 29 weeks for downstream analysis, which led to a total of 561 samples (NEC = 88).

### Diversity analysis based on functional modeling

After considering taxonomy and pathway (Supplementary Figure 2), we next focused on the lowest level of classification obtainable from metagenomics data (i.e., UniRef90 proteins). Since the UniReF90 ≥ 29 weeks PMA were still 4.7 million unstratified proteins, in order to reduce dimensionality and given that there is no previous indication of which microbial feature should be over or under abundant in NEC compared to preterm controls, we used a Kruskal-Wallis test coupled with Bonferroni correction to determine the subset of microbial proteins that were significantly different between NEC and controls. From the Kruskal-Wallis test we selected entries with an adjusted *P* < 0.0001 (Bonferroni), resulting in 3,420 unstratified UniRef90 proteins, which were converted to KEGG orthologs (KO) in a stratified way, meaning that the same ortholog gene could be associated to a different species and kept as a separate feature. Initially, this led to 136,538 unique KO-species feature combinations, of which only 573 had an adjusted *P* < 0.0001 (Bonferroni) between study groups. Critically, 569 KO-species combinations were unique, suggesting there are specific KO-species combinations that differ by disease status that otherwise would be masked by collapsing the data into a single KO derived from multiple species. For exploratory purposes, we used Principal Component Analysis (PCA) derived from a Bray-Curtis dissimilarity matrix to explore the 573 stratified KO-species features set (Figure 1B). Clustering by NEC status was significant on the PCA plot, however with a low effect-size (*P* = 0.001; *R*^2^ = 0.05, Adonis).

### Discriminatory taxa support a known role for proteobacteria

**Figure 2:**
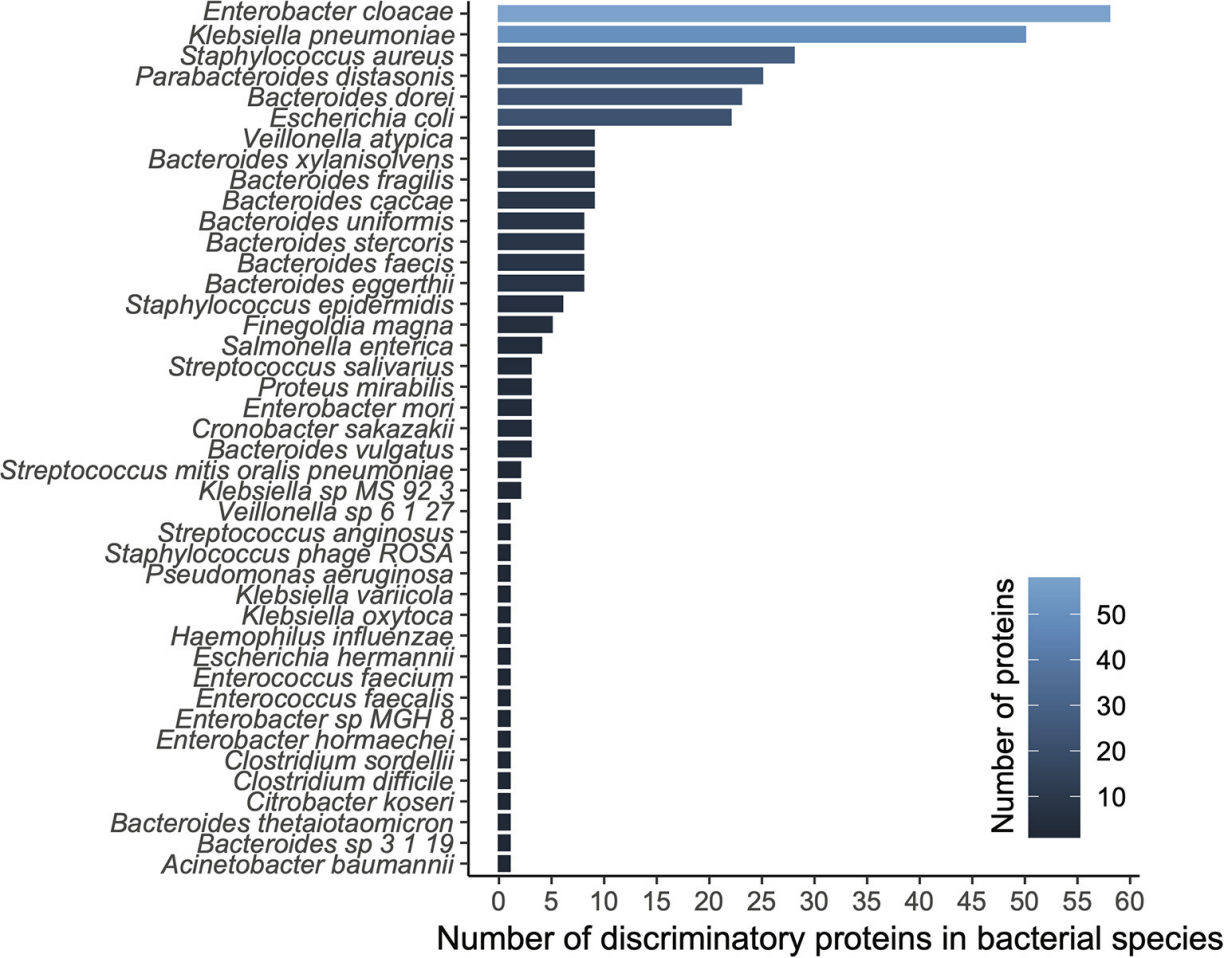
Diversity analysis based on functional modeling. A histogram of proteins grouped by their respective species.

We applied a ranking technique to the significant unstratified UniRef90 entries (*n* = 3,420; see methods) resulting in a hierarchy of 300 highly discriminatory proteins (training accuracy = 0.992; testing accuracy = 0.988) according to disease status. When stratifying by species level taxonomy, these 300 UniRef90 proteins corresponded to 834 features, with 108 unique bacterial species carrying those proteins. We then tested a new Random Forest model trained on this dataset yielding 1.0, 1.0, and 0.8 recall on training, testing and validation datasets, respectively. A list of top bacterial species (*n* = 42) having at least a count of 2 discriminatory proteins is reported in Figure 2.

**Figure 3:**
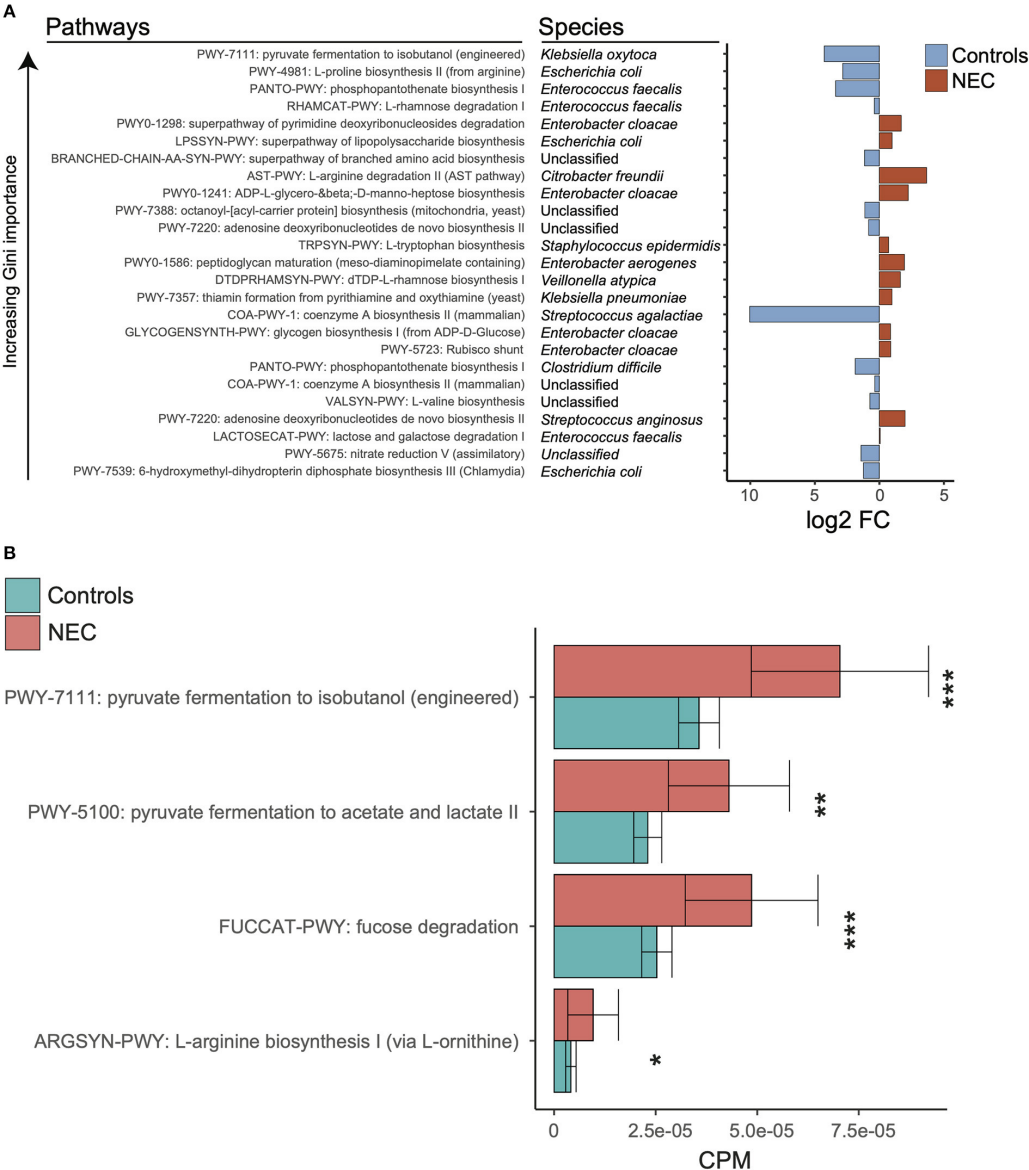
Discriminatory taxa support a known role for proteobacteria. **(A)** Log2 fold change of discriminatory stratified pathways ordered by decreasing gini importance. Red indicates log2 fold change higher in NEC samples and blue indicates log2 fold change higher in control samples. **(B)** Kruskal-wallis tests were run between NEC and control samples for relative abundances of selected pathways from *klebsiella pneumoniae*. The *, **, and *** symbols indicate *P* < 0.01, *P* < 0.01, and *P* < 0.001 respectively.

The top ranked bacterial species were mostly composed of *Enterobacteriaceae*, including the top three ranked species by mean relative abundance, *Enterobacter cloacae* (14% (NEC); 5% (control)), *Klebsiella pneumoniae* (19%; 6%), *and Staphylococcus aureus* (3%; 1%), which were all on average more than 3-fold higher in the NEC cohort compared to controls (Figure 3A). *E. cloacae* harbored the highest number of discriminatory proteins (*n* = 58 or 10%) between NEC and controls, followed by *K. pneumoniae* (*n* = 50 or 8%) and *Staphylococcus aureus* (*n* = 28 or 5%). Taken together, these three species were responsible for carrying 22.8% of the total identified discriminatory proteins; however, most of the UniRef90 proteins (*n* = 269 or 45%), were associated with an uncharacterized taxonomy given the proteins were identified via translated search and thus could potentially belong to multiple species. Importantly, the majority of the species in the top ranking were all taxa associated with previously identified signatures of gut dysbiosis, known to harbor virulence factors as well as antibiotic resistance genes in the early-life microbiome (14, 17). These same species have been shown to contribute to the nosocomial microbiome and therefore potentially colonizing patients at-risk for NEC (8, 15). Conversely, top ranking proteins that were higher in controls were mostly species within the *Bacteroides* genus, which were often undetected in samples from patients with NEC.

### Specific changes in functional pathways are more critical than taxonomy alone

In order to determine the relationship between taxonomy and associated metagenome conferred biologic functions, Mean Decrease in Accuracy was calculated for all features at the pathway level for the top model (stratified pathways, PMA ≥ 29, gradient boosting classifier, and balanced NEC classes). We focused on downstream analysis for the pathway level at ≥29 weeks PMA with stratification in order to discriminate related bacterial species. We selected the top combination of pathway-species based on increasing Gini importance (Figure 3A). Given the importance of microbe and enteral feedings in newborns with NEC, we sought to determine if there were potential metabolic differences associated with the NEC metagenomes that were specific to the identified taxonomic changes. Notably, significantly different pathways at the species levels did not hold significance at the genus level, which reinforced the concept that there is a specific molecular signature through a combination of species and pathways that points to a trend in specific organic acid fermentation pathways (Figure 3B). Specifically, within the *Klebsiella pneumoniae* associated pathways there was a significantly increased number of represented genes in NEC samples compared to controls (Supplementary Table 1). Given these genes govern pyruvate fermentation to isobutanol as well as acetate and lactate it was suggestive of increased metabolic potential for SCFA conversion in the NEC patients compared to controls.

### Metabolic changes associated with metagenomic functional pathways in the newborn fecal stream

**Figure 4:**
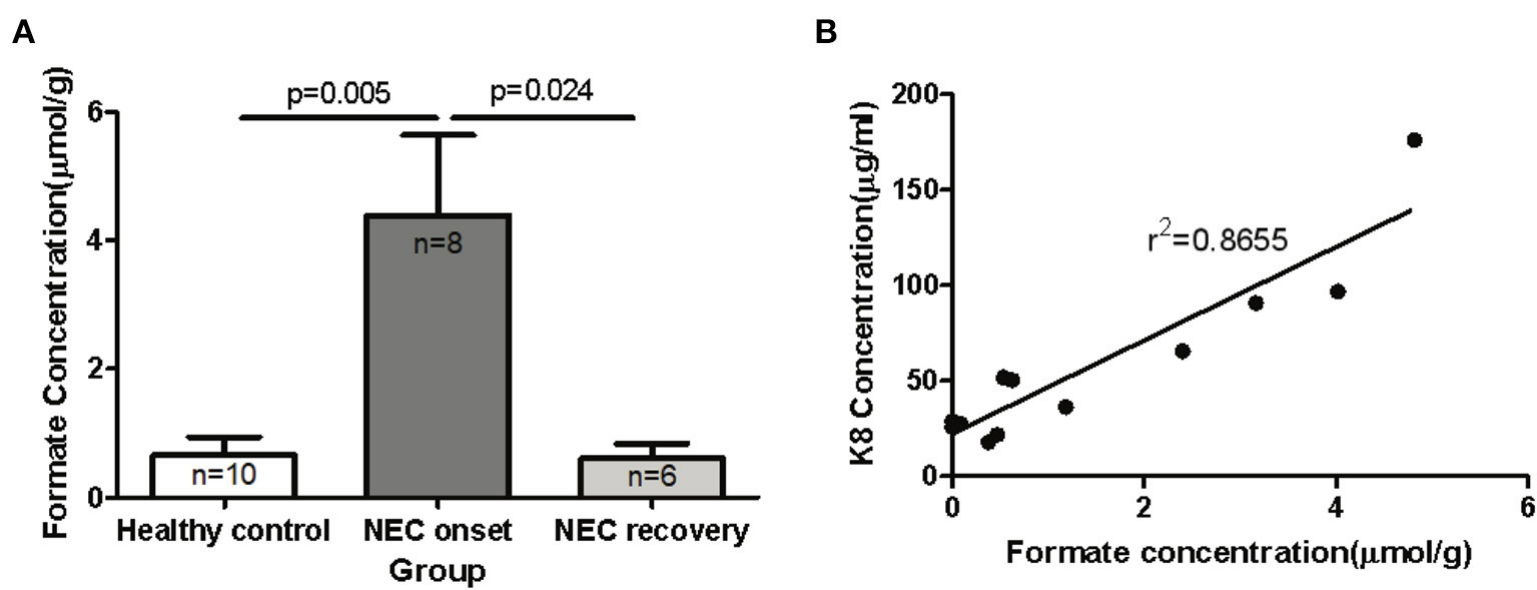
Elevated levels of formic acid (or formate anion) found in fecal specimens of human neonates with NEC. Fecal samples were collected from human preterm neonates and used for mass spectrometric SCFAs assay and K8 ELISA assay. **(A)** The comparison of fecal formate in control and NEC patients (onset and recovery) was performed with unpaired *t*-test. The concentration of fecal formate is significantly elevated in NEC patients at disease onset compared to non-ill premature newborns and formate levels return to baseline during recovery from NEC (*P* < 0.05). **(B)** The relationship of fecal formate and K8 was analyzed by linear regression test (*r*^2^ = 0.8877).

The ability to degrade and ferment various enteral carbon sources is one of the defining characteristics of competing microbiomes; therefore, we reasoned that the functional metagenomic shifts described above would be associated with significant shifts in major organic acid by-products of microbial fermentation in the gut. In order to identify significant shifts in the newborn fecal stream metabolic pattern uniquely associated with NEC onset and recovery, we quantified organic acids and major SCFAs in fecal samples from non-ill premature infants or those with NEC (Table 1). Intriguingly, we found that the concentration of fecal formic acid (formate) was significantly elevated in NEC patients at disease onset (4.40 ± 3.52 umol/g) compared to gestational aged matched and non-ill premature newborns (0.65 ± 0.90 umol/g; *P* = 0.005) (Figure 4A). Intriguingly, formate levels returned to baseline during recovery from NEC (0.61 ± 0.56 umol/g; *P* = 0.024) and upon resumption of stooling in effected newborns. To determine if changes in fecal organic acid concentrations correspond to degree of intestinal injury, fecal cytokeratin 8 (K8) was measured in the same fecal samples in which the organic acids were analyzed. Since keratin is only expressed by sloughed and or dead enteric epithelial cells (mucosa), K8 is a quantitative biomarker of intestinal injury and epithelial cell specific loss (18). The concentrations of fecal formate strongly correlated (*r*^2^ = 0.8655) with the level of keratin 8 (K8; control group 18.0–65.7 ug/ml, NEC group 90.7–176.0 ug/ml; recovery group 21.7–36.2 ug/ml) indicating the relationship between formate and severity of intestinal epithelial damage (Figure 4B). Given the observation of a significant increase in fecal formate associated with NEC, we re-examined specific KO proteins in pyruvate metabolism and formate production as suggested by the analysis above (Figure 3B). We noted that pyruvate formate lyase (PFL) (KEGG: KO0656; EC 2.3.1.54), the rate limiting enzyme in mixed acid fermentation of pyruvate (**Figure 7**), was 2–3 times more abundant (counts per million, NEC vs. control) in NEC patients than control and was significantly associated with *Klebsiella pneumoniae* (*P* = 0.02; FDR) and *Enterobacter cloacae* (*P* = 3.48 × 10^−7^; FDR), the leading species associated with NEC. Thus, reinforcing the concept that bacterial species-specific functions may represent the defining attributes of *Enterobacteriaceae* dysbiosis in the pathophysiology of NEC.

**Table 1:** Formic acid uniquely elevated in fecal samples of human newborns with NEC.

	**Healthy control**		**NEC on-set**		**NEC recovery**		***p***	***p***
**(μmole/gram of stool)**	**Mean**	**SD**	**Mean**	**SD**	**Mean**	**SD**	**NEC on-set vs. control**	**NEC on-set vs. recovery**
Glycolic acid	1.542	0.464	1.370	0.128	2.704	2.124	0.287	0.185
Lactic acid	4.835	6.366	2.712	2.275	5.525	11.224	0.347	0.570
Formic acid	0.651	0.900	4.399	3.518	0.608	0.559	0.020^**^	0.019^**^
Acetic acid	7.917	3.491	9.413	3.463	9.003	1.975	0.378	0.785
Propionic acid	0.688	0.509	0.964	0.868	1.036	1.356	0.442	0.913
Isobutyric Acid	0.070	0.133	0.001	0.001	0.161	0.190	0.135	0.094
Butyric acid	0.204	0.132	2.089	5.494	0.570	0.765	0.364	0.464
Succinic acid	5.691	10.453	4.273	3.055	4.709	4.821	0.691	0.851
Valeric acid	0.105	0.127	0.136	0.242	0.037	0.010	0.746	0.286
indole-3-lactic acid	0.018	0.058	0.000	0.000	0.000	0.000	0.343	na
indole-3-acetic acid	0.055	0.054	0.019	0.003	0.032	0.025	0.066	0.249
indole-3-propionic acid	0.149	0.057	0.113	0.003	0.115	0.004	0.073	0.307
Hexanoic acid	0.353	0.100	1.160	1.939	0.344	0.064	0.277	0.273
indole-3-butyric acid	0.256	0.026	0.239	0.001	0.240	0.002	0.075	0.273
Pyruvic acid	0.771	0.393	0.714	0.564	0.608	0.311	0.815	0.660

** p < 0.05, statistic significance.

### Formate-induced intestinal epithelial injury and barrier compromise

**Figure 5:**
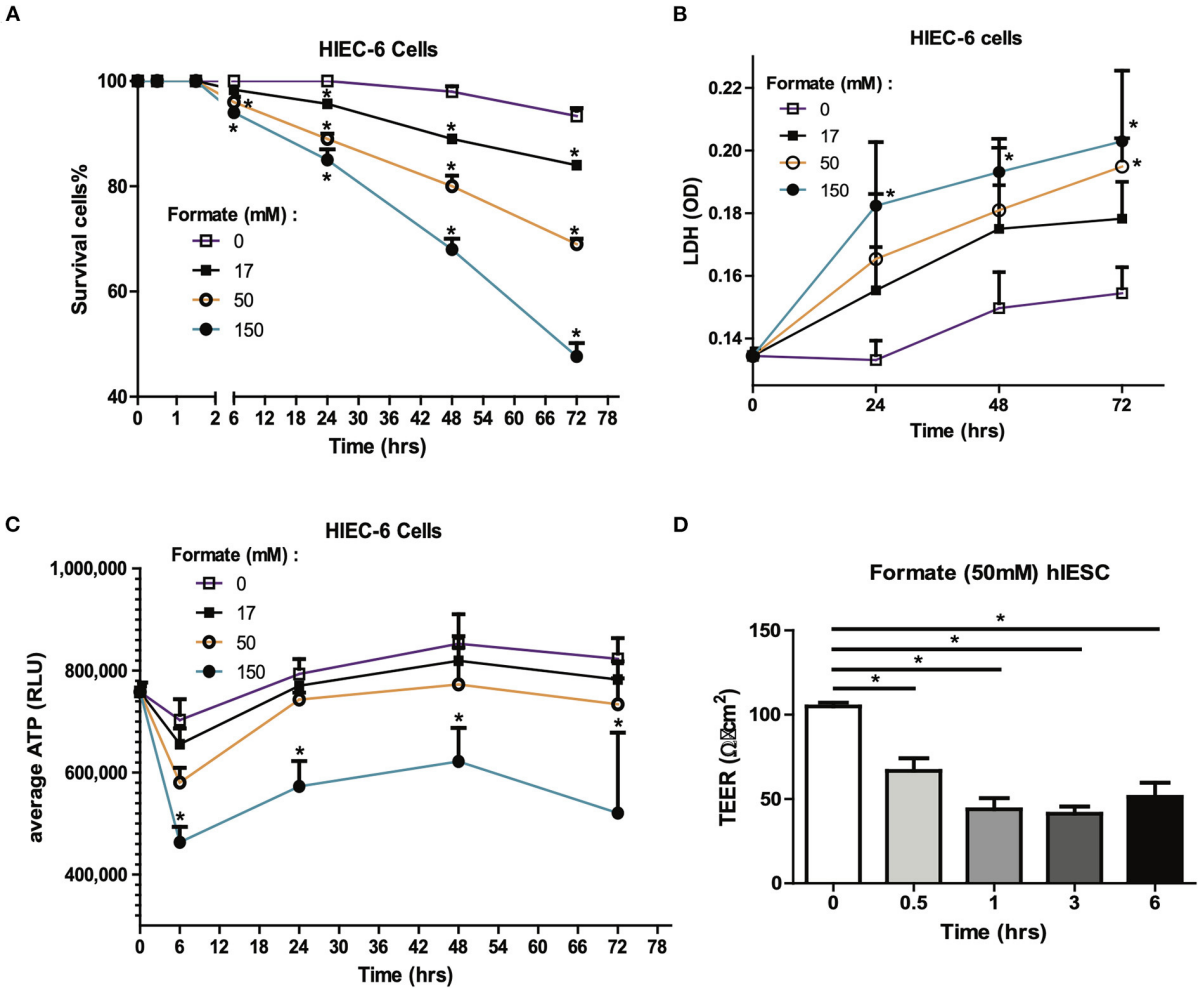
Formate causes intestinal epithelial injury and barrier dysfunction *in vitro*. Effect of Formate on HIEC-6 cells was determined using trypan blue cell viability **(A)** and LDH cytotoxicity assay **(B)** at indicated concentrations and time-points. **(C)** Formate inhibits the production of ATP. **(D)** Formate impairs the integrity of intestinal monolayer epithelial cells derived from hIESCs as measured by permeability. The * symbol indicates the value of *p* < 0.05.

Formate is a common bacterial fermentation end-product liberated upon pyruvate conversion to acetyl-CoA by pyruvate formate lyase (PFL). Increased formate end-product has been associated with *Enterobactereaceae* predominant bacterial blooms in human colitis and mouse models of inflammatory colitis (19). As many fermentation products are produced by colonizing bacteria, many of these organic acids are either processed by the host typically as a fuel source or potential mediator, or conversely further oxidized by specific *Enterobacteriaceae* carrying terminal oxidases in the presence of increased mucosal oxygen present during colonic inflammation. Accordingly, we sought to determine the effects of formate on intestinal epithelium independent of the presence of potentially oxidizing microbes. Here, the cellular effects of formate on human intestinal epithelial cells (HIEC6) were examined in a dose and time dependent manner. Specifically, enterocyte exposure to sodium formate *in vitro* resulted in a dose dependent and significant increase in cell death as early as 6 h with toxic effects continuing cumulatively for up to 72 h at all tested doses (17, 50, and 150 mM). The cellular response was quantified as cell survival rate (Figure 5A) and cytotoxicity as measured by lactate dehydrogenase (LDH) release (Figure 5B). Importantly, the toxicity effect appeared to be specific to formate as parallel studies using common fermentation organic acid products in the newborn gut, including acetic acid (AA) and lactic acid (LA), did not result in any measurable cellular toxicity in contrast to that observed with formate (data not shown).

Given the known mechanism of formate in un-coupling the electron transport chain with consequent effects on cellular respiration (20, 21), cellular ATP production was serially measured in HIEC-6 cells in response to formate (Sodium Formate) exposure. A significant reduction in cellular ATP production was observed with the most pronounced to 65.9% of control group (*P* = 0.0018) at 6 h of exposure at 150 mM of formate (Figure 5C). Since a reduction in gut barrier integrity has been critically linked to NEC onset (22, 23), we next tested the effect of formate on gut barrier function using monolayer HIEC6 cells and transepithelial electrical resistance (TEER) testing. Here, we observed a significant reduction in gut barrier function from 105.0 to 66.7 Ω • cm^2^ within 30 min of exposure (*P* = 0.0012) with continued compromise (51.3 Ω • cm2; *P* = 0.0003) for up to 6 h of exposure to 50 mM of formate, which corresponded to the early peak reduction in ATP production (Figures 5C,D). ATP reduction and barrier integrity disruption were also associated with a significant reduction in phosphorylated AKT and increased phosphorylated-mixed lineage kinase domain-like protein p-MLKL (Supplementary Figure 3), known to promote necroptosis-induced inflammation and loss of enterocyte integrity (24, 25). Taken together, these data suggest a mechanism in which formate can induce necroptotic cellular death supportive of previous observations associated with NEC (25).

### NEC-like intestinal injury in newborn mice after formate treatment

**Figure 6:**
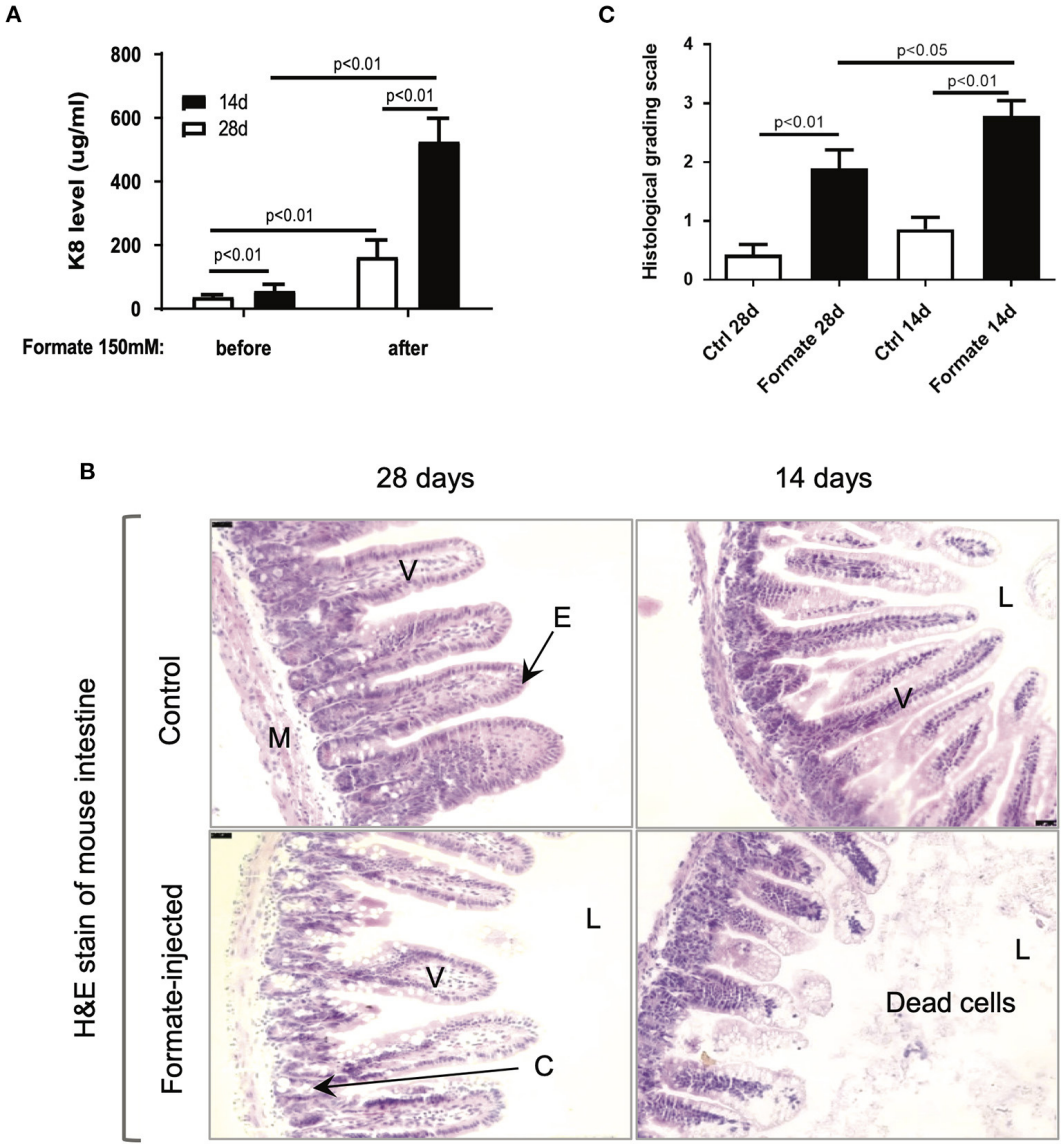
Formate causes intestinal epithelial injury *in vivo*. **(A)** Luminal administration of formate induces intestinal epithelial injury of neonatal mice in an age-dependent manner as 14-day mice and 28-day mice demonstrate a differential response to the same challenge (*n* = 8 in each group; Paired *t*-test was performed). **(B)** H&E stain of mouse intestines that were treated with or without 150 mM of Formate (V, villus; E, epithelial cells; M, muscle layer; L, lumen; C, crypt). **(B)** Histological grading scale of mouse intestinal epithelium; [unpaired *t*-test). Ctrl, control]. **(C)** Histological grading scale of mouse intestinal epithelium; (unpaired *t*-test) Ctrl, control.

In order to test the effects of formate on intestinal integrity *in vivo*, a mouse model comparing weaning (14 day) and weaned (28 day) newborn mice were utilized. Prior publications have described the developmental stage-dependent changes in mouse gut morphology and function upon comparison of 14d and 28d mouse testing (26–29). Accordingly, 14d and 28d mice underwent direct intraluminal injection of formate into the terminal ileum in order to mimic the distribution of NEC injury at one of two different doses (150 and 300 mM) and were euthanized for assessment at 24 h of exposure. A significant dose and developmental stage-dependent gut injury response was observed at 24 h (Figures 6A,B). Notably, the degree of injury, as determined by total fecal K8 levels, was markedly and significantly increased in the 14d (56.1 ± 21.1 vs. 525.6 ± 73.3 ug/ml; *n* = 11) compared to the 28d (37.1 ± 7.0 vs. 162.9 ± 53.3 ug/ml; *n* = 8) mouse gut (*P* = 0.00072), respectively (Figure 6A). Specifically, the histological appearance of the ileum in the 14d mouse reflected an overall increased injury pattern, evidenced by significant crypt-villus axis disruption, sloughed mucosa and numerous dead cells with karyolysis, while shedding from villi to the lumen was accompanied by an overall shortening of effected villi (Figure 6B). Blinded pathologic assessment and grading of the epithelial injury pattern according to the previously described NEC score (Supplementary Table 2) (30), revealed a significant increased injury pattern in both 14d (2.79 ± 0.98 after formate vs. 0.86 ± 0.77 baseline, *P* < 0.01) and 28d (1.89 ± 1.10 vs. 0.43 ± 0.65, *P* < 0.01) mouse ileum, with a significant increased overall NEC score in the 14 day mouse ileum (2.79 ± 0.98 in 14d vs. 1.89 ± 1.10 in 28d, *P* < 0.05) reflecting the histologic picture and corresponding to the fecal K8 levels (Figure 6C).

## Discussion

**Figure 7:**
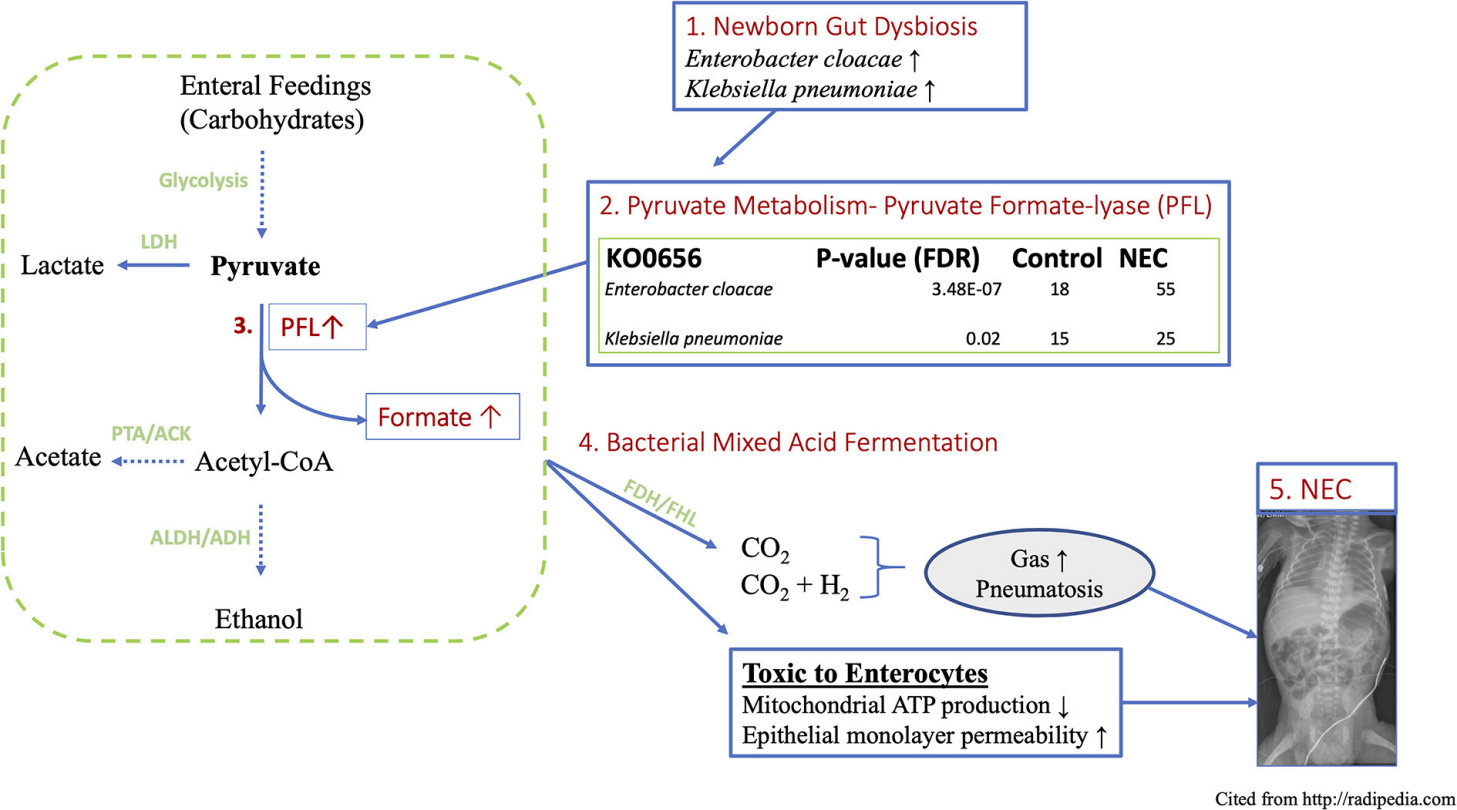
Schematic working model of proposed novel mechanism of NEC induced by gut functional dysbiosis in human premature newborns. (1) Functional metagenomic analysis revealed that *Enterobacter cloacae* and *Klebsiella pneumoniae* are the most discriminatory taxa related to NEC dysbiosis, (2) These top-two species are major contributors to the KEGG ortholog KO0656, *pyruvate formate lyase*, which directs the bidirectional conversion of pyruvate to Acetyl-CoA and formate. (3) PFL is up to 3-folds higher in NEC vs. control. (4) The concentration of formate increases more than 6-folds in fecal samples of NEC patient via a key anaerobic pathway of mixed acid fermentation. (5) Excess fermentation products including formate and gas produce epithelial cytotoxicity and the hallmark sign of *pneumatosis* intestinale observed in intestinal mucosal injury in NEC. Parts enclosed in rectangular boxes are direct results from current study. LDH, Lactate dehydrogenase; PFL, Pyruvate formate lyase; ALDH, aldehyde dehydrogenase; ADH, alcohol dehydrogenase; PTA, phosphotransacetylase; ACK, acetate kinase.

The present study is the first to our knowledge to demonstrate a link between premature newborn gut dysbiosis, the acquired newborn disease Necrotizing Enterocolitis (NEC) and a mechanism of biochemical mediated intestinal injury conferred through microbial fermentation and the over-production of formate (Figure 7). This work extends prior observations by explaining the biochemical consequences of the documented wide-spread Proteobacteria predominant gut colonization patterns preceding NEC in NICU hospitalized premature newborns (5, 11, 26). The functional metagenomic modeling herein provides evidence to support the previously identified 29–32-week post-menstrual age window of peak NEC susceptibility and onset (4). Specifically, the observation of significantly enriched *Enterobacteraceae* species and associated metabolic pathways resulting from their respective genomes in premature newborns with NEC is most pronounced in the designated PMA range providing a plausible biological explanation for this heretofore unexplained observation. Moreover, increased formate production quantified in the stool of newborns with NEC along with the cellular and intestinal injury caused by formate through the arrest of cellular respiration and gut barrier disruption provide a pathophysiologic mechanism of action linking the dysbiotic gut with intestinal injury patterns observed in premature newborns with NEC. Taken together, these findings implicate aberrant colonizing microbe metabolism as the pathophysiologic link between Proteobacteria colonization and NEC in preterm newborns.

In the gut, colonizing bacteria compete for carbon sources to establish their respective ecological niches. Optimization of carbon sourcing for colonizing bacteria is dependent on the human newborn host to provide fermentable substrate in the form of protein and carbohydrate food substances. The mutualism model and the provision of microbe directed ecological services suggests the process of human newborn gut colonization has evolved to maximize synergistic benefits between human host and gut microbiota (31). A full 15% of volume content of the infant's first food substance, human breast milk (HBM) is composed of human milk oligosaccharides (HMOs) that cannot be digested by newborns. HMOs are the natural source of fermentable polysaccharide for human newborn colonizing bacteria and are therefore matched to the selection of specific microbial species that are genetically endowed to fully consume this prebiotic (32, 33). Accordingly, one of the primary benefits of HBM can be viewed as providing the needed selection pressure for health-promoting favorable microbes like bifidobacteriae (27, 34) that carry the necessary molecular capacity for HMO metabolism (33, 35). In the absence of favorable HMO consuming colonizers reduced through a variety of detrimental mechanisms including pervasive perinatal antibiotic usage in NICU hospitalized newborns, there is a taxonomic shift in colonizing bacteria from the obligate anaerobic phyla *Bacteroidaceae* and *Firmicutes* toward facultative anaerobic Proteobacteria that should typically represent a minority population in newborns and health adults (36). Thus, the ability to ferment and degrade various host diet-derived carbohydrate is a major determinant of successful gut microbial colonization but requires the presence of a capable symbiont that might have been placed under strong de-selection pressure through medical interventions in the NICU.

The balance among various obligate anaerobes is dramatically disrupted in the gut of most NICU hospitalized newborns, representing an archetype dysbiotic state with a shift toward facultative anaerobic and over representation of specific species within the *Enterobacteraceae* family (e.g., *Klebsiella pneumoniae, Enterobacter cloacae*) (8, 10, 37, 38). This dysbiotic state has been established as inflammation inducing (10, 15), along with the potential for profound changes to the metabolite milieu resulting from the alternative modes of nutrient degradation and macromolecule fermentations (39, 40). Indeed, several publications have shown a similar pattern of proteobacteria predominant colonization patterns in NICUs broadly (8, 15) and more specifically in preterm babies developing the gut pathology of NEC (41).

The complexity of the human intestinal microbiome and widespread dysbiosis among NICU hospitalized newborns has confounded the search for a pathophysiologic connection between changes in taxonomy and NEC onset (8, 15). To probe the functional relationship between NEC and enteric dysbiosis in premature newborns, we assembled and analyzed publicly available metagenomic sequencing of newborns that developed NEC and age-matched preterm newborns that did not. To extend prior analyses we examined species level differences and associated metabolic pathways that suggested specific alterations conferred by the representative microbes and their functional consequences. The functional metagenomic findings herein strongly implicate the *Enterobacteraceae* species *Enterobacter cloacae* and *Klebsiella pneumoniae* as major contributors to the KEGG ortholog KO0656, *pyruvate formate lyase*, which directs the bidirectional conversion of pyruvate to Acetyl-CoA and formate. This biochemical mechanism connects the species-specific *Enterobacteriaceae* signature and bacterial formate production as a plausible causative factor involved in mucosal injury observed in NEC. Indeed, our analyses demonstrate that these two specific microbial species that are strongly over-represented in NEC contribute over 90% of the microbiome contribution to that gene (Figure 7).

Indeed, machine learning models as well as classic statistical approaches computed at different levels of the microbiome including taxonomy, pathways and genes, all pointed to the same microbial signatures as the most powerful discriminatory feature between NEC and control groups. It is apparent that the approach presented herein of refined species-specific gene and pathway stratification rather than the collective orthologous functions of all microbes, provides the highest predictive power and possible pathophysiologic resolution when considering the role of the microbiome in NEC onset. Changes in fecal stream metabolites represent the functional interaction of the species-specific microbiome, the host and enteral substrates. Organic acids represent intermediate substrates in gut fermentation processes with observable effects on gut fecal pH (lactate), substrates for enterocyte fuel sources (e.g., butyric, lactic, and acetic acid) and intermediates of microbial and host respiration. Intriguingly, among a panel of measured common organic acids in human newborns during NEC onset, we observed a dramatic and significant increase in enteric formate production as measured in the fecal stream. The meaningfulness of these observations was reinforced by the temporal association with NEC onset and the return to baseline of formate during NEC resolution to levels similar to non-NEC control infants. Since formate has been linked to microbial respiration associated with an expansion of Proteobacteria and enteric inflammation (19), formate appears to fulfill criteria as a functional mediator of intestinal injury arising from premature newborn dysbiosis.

Finally, in order to further investigate whether a lead pathogen-derived metabolite would fit within the development dependent toxicity model of NEC described here and previously (25, 28), we empirically tested formate in a rodent model of enteric injury at developmentally disparate times chosen to mimic NEC. We found that formate indeed produces a dose-dependent toxicity to enteric epithelia in both human cell model(s) and pre-clinical rodent models. Taken together, metagenome modeling and metabolic toxicity testing suggest that a microbial signature that is highly accurate in defining NEC onset likely represents the intersection with a susceptible and exposed host enteric mucosa. The emerging metabolic model of NEC presented herein implicates significant differences in pyruvate metabolism resulting in an over-abundance and accumulation of formate in the gut of human newborns that temporally coincides with NEC onset and resolution. We speculate that the emerging metabolic model of NEC herein may accommodate the variable rates of NEC in similarly dysbiotic newborns given variability in formate production as a combinatorial function of intestinal transit, enteral substrate, microbial composition, and enteric susceptibility to injury.

In conclusion, the multi-omics approach used in this study suggests a novel mechanism of NEC onset through the production and accumulation of enteric fermentation products, particularly formate. This metabolic model of NEC is consistent with and extends prior work suggesting newborn enteric microbial community adaptation of specific members as a first step toward the development of NEC (42). *Enterobacter cloacae* and *Klebsiella pneumonieae* were the most discriminatory taxa related to dysbiosis and increased formate production which elicits a dose-dependent toxicity to enteric epithelia consistent with NEC-like intestinal injury. These findings support ongoing efforts to prevent NEC through the reduction of newborn gut dysbiosis including decreasing the abundance of specific risk-associated taxa and providing possible metabolite biomarkers for determining the effectiveness of interventions including early-life probiotic feeding with a determination of effect on enteric biochemical changes.

## Materials and methods

### General study design

This study was conducted in several stages as follows: (I) *In silico* aggregation and modeling of premature newborn metagenomes in NEC and post-conceptual age-matched newborns; (II) Determination of targeted organic acid concentration changes in the fecal stream of NEC (cases) and PCA matched premature newborns; (III) SCFA toxicity testing *in vitro* and *in vivo*.

### Cohort selection and data extraction

To probe the functional relationship between NEC and enteric dysbiosis in the premature newborn, we assembled and analyzed publicly available metagenomic sequencing data of newborns who developed NEC along with age matched preterm newborns that did not develop NEC. A total of eight studies that performed shotgun metagenomic sequencing matching the word “NEC” or “preterm” on NCBI Sequence Read Archive (SRA) were chosen for analysis. A detailed summary of the studies, sequencing count and patient characteristics can be found in Supplementary Table 3. In order for a sample to be included in the analysis a minimum of intrinsic metadata criteria had to be met in regard to reporting “day of life,” “NEC presence/absence,” “antibiotic treatment,” “country of origin,” “gestational age,” “delivery mode,” “feeding practice,” “sex,” and “birth weight.” After applying filtering criteria based on meta data, a total of 1,647 shotgun metagenomic raw datasets were retained. These represent every shotgun metagenomics sequencing dataset from preterm babies available in the NCBI SRA as of September 2019.

### Feature annotation

Samples were analyzed concurrently within the same computational pipeline. Taxonomic profiling of the metagenomic samples was performed using MetaPhlAn2 (43) with default parameters, using the included library of clade-specific markers to provide panmicrobial (bacterial, archaeal, viral, and eukaryotic) profiling. Functional gene characterization was performed using the HUMAnN2 (44) pipeline with default settings following the updated global profiling of the Human Microbiome Project analysis pipeline (45). After MetaPhlan and HUMAnN2 pipeline, several different matrices were obtained containing taxonomic or functional annotations based on different classifications against UniRef90 (46), KEGG (47), and MetaCyc (48) databases.

### Data preparation and feature selection

A taxonomic relative abundance matrix created by HUMAnN2 was obtained and a target post-conceptual (gestational age plus postnatal day) age window was obtained through statistical learning techniques. Random forest models were trained to predict NEC from taxonomic relative abundances on progressively increasing windows of PMA (full window and shifting windows of 2, 3, 4, and 5 weeks of PMA and ages of outlying samples were filtered out). Furthermore, false positives and false negatives from the above models were analyzed by gestational age to reveal trends between PMA and incorrectly classified samples. The full pipeline is outlined in Supplementary Figure 2.

### Pathway identification and ranking through machine learning

An initial two datasets, an unstratified pathway abundance dataset and a pathway abundance dataset stratified by bacterial species, were divided into two groups: those with PMA <29 and those with PMA ≥ 29. Each of these two datasets was further divided into four smaller datasets: a training set with original NEC distribution (unbalanced), a training set with oversampled NEC distribution (balanced), a testing set (20%) of unique samples, and a validation set (20%) of unique samples. For each training dataset, a Gradient Boosting Classifier from python's scikit-learn library was trained to classify each sample as NEC or non-NEC. Hyperparameters were grid-searched for each dataset and the model with highest specificity and sensitivity was selected for further analysis.

Feature importance was calculated from the highest performing hyperparameters using Gini importance scores. Because Gini importance scores account for the impurity at each node, these scores were expected to change significantly between the balanced and unbalanced datasets. Thus, to confirm findings from feature importance scores, ranking was also conducted. Pathway feature ranking was determined by conducting recursive feature elimination with a step of 50 on a random forest classifier. Subsequently, the top 500 features were ranked implementing recursive feature elimination on the same model with a step size of 1.

### Statistical analysis and ranking of 90% identity proteins

Significantly different genes among groups were estimated using the Kruskal–Wallis one-way analysis of variance, coupled with FDR or Bonferroni correction as cross-sample normalization. A Bray-Curtis dissimilarity matrix was constructed to estimate global differences among samples and visualized via Principal Coordinate Analysis (PCoA). Permutational Multivariate Analysis of Variance Using Distance Matrices (adonis) was used to assess global microbiome differences between treatments and the effect-size (*R*^2^) of disease status on the microbiome composition *P*-value for PCoA panel was computed using F-tests based on sequential sums of squares from permutations of the raw data. *P*-values throughout the manuscript are represented by asterisks (^*^P < 0.05; ^**^P < 0.01; ^***^P < 0.001; ^****^P < 0.0001).

The sub list of significant proteins was further filtered to eliminate UniRef90 ids associated with “obsolete” proteins yielding a total of 1,815 proteins. Protein feature ranking of UniRef90 proteins was determined by conducting recursive feature elimination with a step of 1 on a random forest classifier. To determine the minimum number of top ranked features to yield high accuracy and generalizability, a random forest classifier was trained on the top features from top 1 to top 746. A train, test, and validation accuracy score was calculated for each set of top ranked features. The associated taxa from these proteins were obtained from HUMAnN2 output files and these were analyzed to determine which species were associated with NEC. A final random forest model was trained and ranking conducted on these proteins stratified by species to confirm the model and increase understandability of the previous model. The workflow for the random forest and ranking were identical to the first (unstratified) protein model.

### Short chain fatty acids (SCFAs) analysis

**Twenty-four** fecal specimens were collected from 10 non-ill age-matched premature newborns and 3 patients with NEC, 8 stools at disease onset and 6 stools during recovery and after 3 weeks' clinical treatment. A panel of SCFAs (Table 1) were quantified using Agilent 6490 triple quadruple mass spectrometer following extraction from fecal samples as previously described (49). Briefly, samples were diluted with 80% ethanol (10 μl/mg) and gently agitated overnight at 4°C. The homogenized samples were centrifuged at 21,000 × g for 5 min. 200 μl of the supernatants were transferred centrifuged at 21,000 × g again for 20 min. For each sample, 20 μl of the supernatant was mixed with 20 μl of 200 mM N-(3-Dimethylaminopropyl)-N′-ethylcarbodiimide hydrochloride (1-EDC HCl) (SIGMA) in 5% pyridine (SIGMA) and 40 uL of 100 mM 2-Nitrophenylhydrazine (2-NPH) (SIGMA) in 80% acetonitrile (ACN) (SIGMA) with 50 mM HCl. The mixture was incubated at 40°C for 30 min. After reacting, 400 μl of 10% ACN was added to the solution. Then 1 μl the solution was injected into an Agilent 6490 triple quadruple mass spectrometer for analysis.

### Cell culture assays

The human intestinal epithelial cell line (HIEC-6) was acquired from the American Type Culture Collection (ATCC, Manassas, VA, USA) and cultured in opti-MEM medium (Gibco) with 4% FBS (Gibco), 10 ng/mLEGF (Gibco), and 1% penicillin-streptomycin. Small intestinal samples were obtained fresh from the Surgical Pathology Department of Stanford University. The 24-well Transwell inserts (Corning 3414) were coated with collagen IV (30 μg/mL) and incubated in 37°C for 2 h to ensure successful coating. The enteroids containing Matrigel buttons were scraped off from the tissue culture plate and disassociated into single cells. Cell containing solutions were then pass through 40 μm cell strainer before centrifuging at 1,000 rpm for 5 min. Cell pellets were resuspended with enteroid culture media supplemented with Y-inhibitor and CHIR-99021, and previously described (50, 51) and seeded onto the apical side of the collagen IV coated Transwell inserts at a density of 750 k/cm2. Monolayers were then cultured in enteroid culture media for 5 d with media changes every 2 d. On day 5, the culture media was switched to differentiation media for 4 d to induce cellular differentiation. The differentiation media was enteroid culture media in the absence of Wnt3a, r-spondin, Nicotinamide and SB-202190. Upon withdrawal of these factors, the proliferative cell lineage is noted to differentiate into a secretory cell lineage (50–57). All cells were cultured in an incubator maintained with 5% CO2 at 37°C.

HIEC-6 Cells (1 × 10^5^/well) were treated with Sodium Formate (Formate) (0, 17, 50, 150 mM) in 48-well plates and stained with Trypan blue solution after 0.5, 1.5, 6, 24, 48, 72 h.

### Cell toxicity and survival assessment

The percentage of surviving cells was calculated as follow. HIEC-6 cells (1 × 10^5^/well- 24 well plate) were seeded and maintained as above. After 70% confluence, the cells were treated with Formate (0, 17, 50, 150 mM). After 24, 48, and 72 h of incubation, 50 μl of the supernatant was collected in triplicate to a 96-well plate and stored in −20°C refrigerator for LDH assay. 50 μl of 2% NP40 were added to each well of the 96-well plate, mixed with the supernatant and incubated at room temperature with shaking for 15 min. One hundred microliter of the Cytotoxicity Detection Kit LDH solution (Takara, Japan) was added to each well. After 15 min of incubation at room temperature in the dark, the OD value was measured at 492 nm in a SpectraMax® i3x multi-mode microplate reader (Molecular Devices, LCC. CA, USA) (620 nm as reference).

The CellTiter-Glo® 2.0 reagent was used for determination of ATP content according to the manufacturer's instructions (for G9242 from Promega Biotechnology Company, Madison, WI). Briefly, 100 μl of CellTiter-Glo® 2.0 Reagent were added to each well. The contents were mixed for 2 min on an orbital shaker to induce cell lysis, followed by incubation at room temperature for 10 min to stabilize the luminescent signal. The plates were read using the SpectraMax® i3x multi-mode microplate reader (Molecular Devices, LCC. CA, USA).

### Transepithelial electrical resistance (TEER) assay

To examine the effect of formate on human intestinal epithelial monolayer permeability, TEER was measured using a EVOM2 Epithelial Voltohmmeter (World Precision Instruments; Sarasota, FL). Monolayers were prepared from differentiated primary small intestine stem cells and cultured in 24-well Transwell inserts prior to treatment with formate (0, 17, 50, 150 mM) at the basolateral side of the monolayer. The TEER measurement was performed at the indicated time points after formate treatment.

### *In vivo* NEC mouse model

FVB/N mice aged 14 d or 28 d (*n* = 20/group) were chosen to mimic human newborns and infants for comparation of their response to intraluminal formate challenge. The mouse abdomen was entered via midline laparotomy with a 1.5 cm incision. After exposure of the ileocecal junction, a sterile solution of sodium formate (150 mM, 10 ul/g body weight) was injected directly into the intestinal lumen using a 27G needle toward the proximal direction. The abdominal wall was closed in layers using 4-0 vicryl suture. Stool sample pellets were collected individually for 24 h and stored at −80°C. After 24 h, mice were sacrificed, and small intestines harvested and stored either at −80°C or formalin-fixed. All experiments were conducted under a protocol approved by Stanford University School of Medicine Institutional Animal Care and in accordance with NIH guidelines.

### Histological analysis

Intestinal tissues were fixed in 4% paraformaldehyde, embedded in paraffin and 8 μm thick sections were stained with hematoxylin-eosin (H&E) (ScyTek Laboratories, Inc., Logan, UT, USA) according to a standard protocol.

### Evaluation of intestinal injury

A modified ELISA was used to measure the insoluble protein cytokeratin 8 (K8) in stool from both human and mice. Briefly, 96-well MaxiSorp™ Nunc-immuno module plates (Thermo Fisher Scientific) were pre-coated with protein K8 overnight at 4°C. Twenty five microliter of diluted fecal samples and standards were mixed with 25 μl blocking buffer (5% skim milk/PBST/2% NP40) for 30 min with shaking. Fifty microliter anti-K8 antibody (MilliporeSigma, USA) was added to each well and incubated at RT for 1 h with shaking. The mixed samples with antibody were transferred to pre-coated plates and incubated for 1 h at RT with shaking (100 μl/well), followed by washing 3 times in PBST buffer. Hundred microliter HRP (horseradish peroxidase)-conjugated goat anti-rat antibodies (Immnuoreagents lnc.) was added, followed by standard ELISA procedures. The plates were read at 450 nm with reference filter at 620 nm using the SpectraMax® i3x multi-mode microplate reader (Molecular Devices, LCC. CA, USA). Final concentrations were obtained using the standard curve method.

### Western blot analysis

Whole protein lysates from mouse intestinal tissues were prepared using RIPA lysis buffer (Thermo Fisher Scientific, Rockford, IL, USA). Samples were loaded into 10% sodium dodecyl sulfate–polyacrylamide gel electrophoresis (SDS-PAGE) gels (BIO-RAD, Hercules, CA, USA), transferred to polyvinylidene difluoride membranes (Amersham Biosciences, Buckinghamshire, UK) and incubated with primary anti-K8 antibody (Troma-1, MilliporeSigma, USA,1:500), antiPGC-1α (Abcam, Cambridge, MA, USA, 1:500), anti-phospho-MLKL (p-MLKL) (CST, 1:500), anti-AKT (CST, 1:500), anti-cleaved caspase 3 (CST, 1:500), anti-GAPDH (CST, 1:1,000) and anti-cleaved-K18 (AnaSpec, Inc., Fremont, CA, USA, 1:1,000) overnight at 4°C. Membranes were incubated with secondary monoclonal goat anti-rat antibody (Immunoreagents, Inc., Raleigh, NC, USA, 1:1,000) or goat anti-rabbit antibody (CST, 1:1,000) at room temperature for 1 h. Protein bands were visualized using an ECL detection kit (Thermo Fisher Scientific, Rockford, IL, USA) according to the manufacture's protocol.

### Statistical analysis of *in vitro* and *in vivo* empirical testing

Data are presented as means ± *SD* and statistical analysis was performed using Student's *t* test (SPSS Statistics, version 19.0, Chicago). *P* < 0.05 was considered statistically significant. The data graphs were made with GraphPad Prism 5.0 software (Graph-Pad Software, CA, USA).

## Data availability statement

The original contributions presented in the study are included in the article/Supplementary material, further inquiries can be directed to the corresponding author/s.

## Ethics statement

The animal study was reviewed and approved by Stanford University School of Medicine Institutional Animal Care.

## Author contributions

GC, SF, BH, and KS conceived the study. GC, SF, BH, G-ZT, JW, and KS designed the study. JW, JL, KW, G-ZT, and P-YL generated data presented in the manuscript. GC, SK, G-ZT, JW, JL, KW, SF, and KS analyzed the data. GC, BH, JD, and KS wrote the manuscript. All authors approved the final manuscript for publication.

## Funding

This work was supported by Evolve Biosystems.

## Conflict of interest

Authors GC, SK, and SF are former employees of Evolve Biosystems. Author BH is currently an employee of Evolve Biosystems. Author KS became a paid consultant to Evolve Biosystems, Inc., and subsequent to completion of this work. The remaining authors declare that the research was conducted in the absence of any commercial or financial relationships that could be construed as a potential conflict of interest.

## Publisher's note

All claims expressed in this article are solely those of the authors and do not necessarily represent those of their affiliated organizations, or those of the publisher, the editors and the reviewers. Any product that may be evaluated in this article, or claim that may be made by its manufacturer, is not guaranteed or endorsed by the publisher.

## Supplementary material

Supplementary Figure 1 Plot of taxonomy model test sensitivity (y-axis) according to PMA (x-axis) (post menstrual age). The week group refers to the range of ages used to train the model(s).

Supplementary Figure 2 Schematic of the metagenomic data modeling strategy.

Supplementary Figure 3 Western blot showing relevant protein effector protein changes in dose response to formate by HIEC-6 cells (human intestinal epithelial cells 6). (mM = milli-molar), AKT = protein kinase B) [pMLKL = phospho-mixed lineage kinase domain-like protein (MLKL) (PGC1a = peroxisome proliferator-activated receptor gamma coactivator)-1alpha].

Supplementary Table 1 Relative abundance of species by sample displayed such that each sample sums to 1.

Supplementary Table 2 Histological grading scale for neonatal mouse model of NEC.

Supplementary Table 3 Sample metadata from publicly available datasets combined into single sheet.
