# Supplementary figures and images for: Metabolic model of necrotizing enterocolitis in the premature newborn gut resulting from enteric dysbiosis
Source: Front Pediatr. 2022 Aug 23;10:893059. doi: 10.3389/fped.2022.893059 (PMC9445129; doi:10.3389/fped.2022.893059)

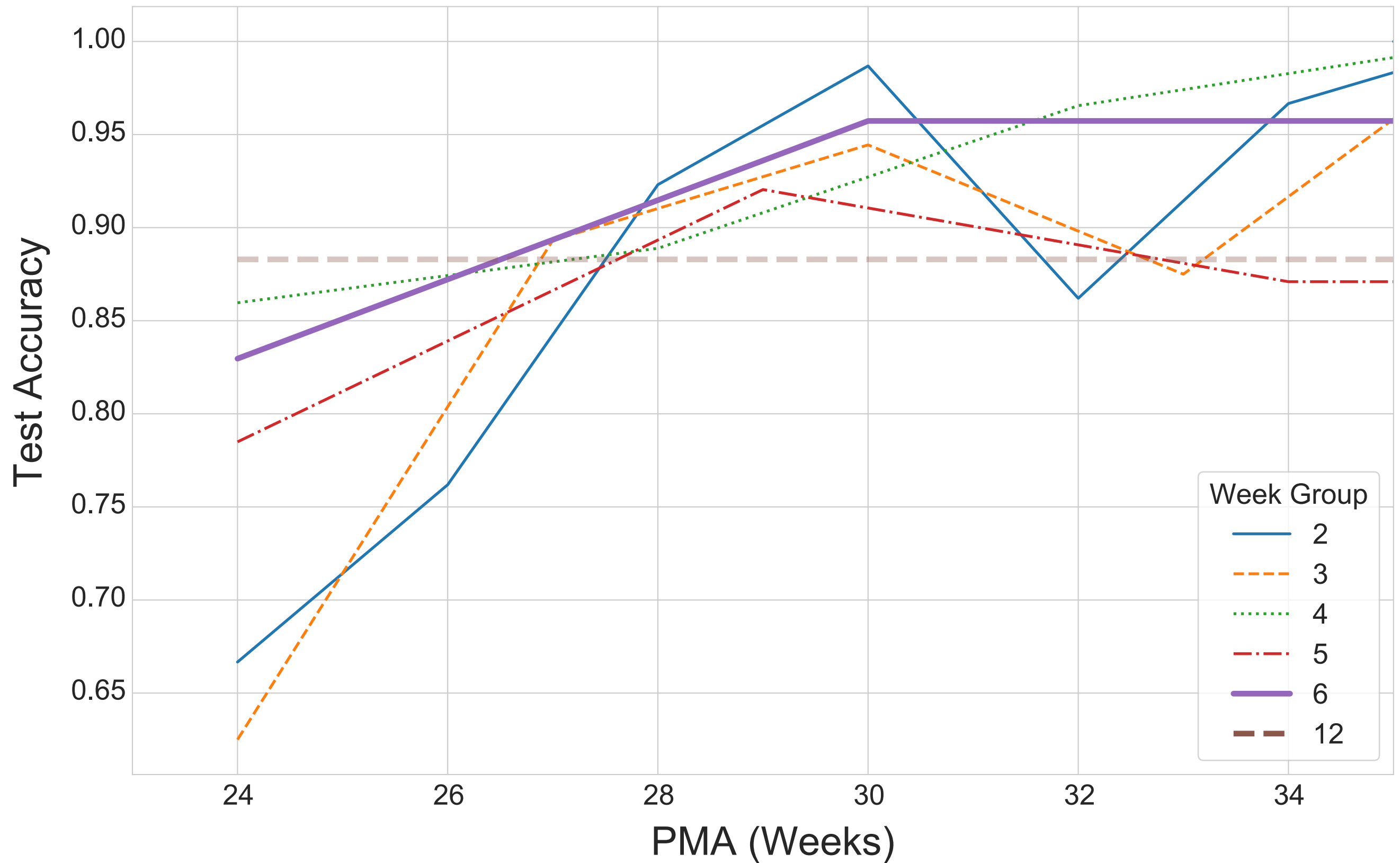

Supplement: Supplementary Figure 1 — Plot of taxonomy model test sensitivity (y-axis) according to PMA (x-axis) (post menstrual age). The week group refers to the range of ages used to train the model(s). [file Data_Sheet_1.PDF]

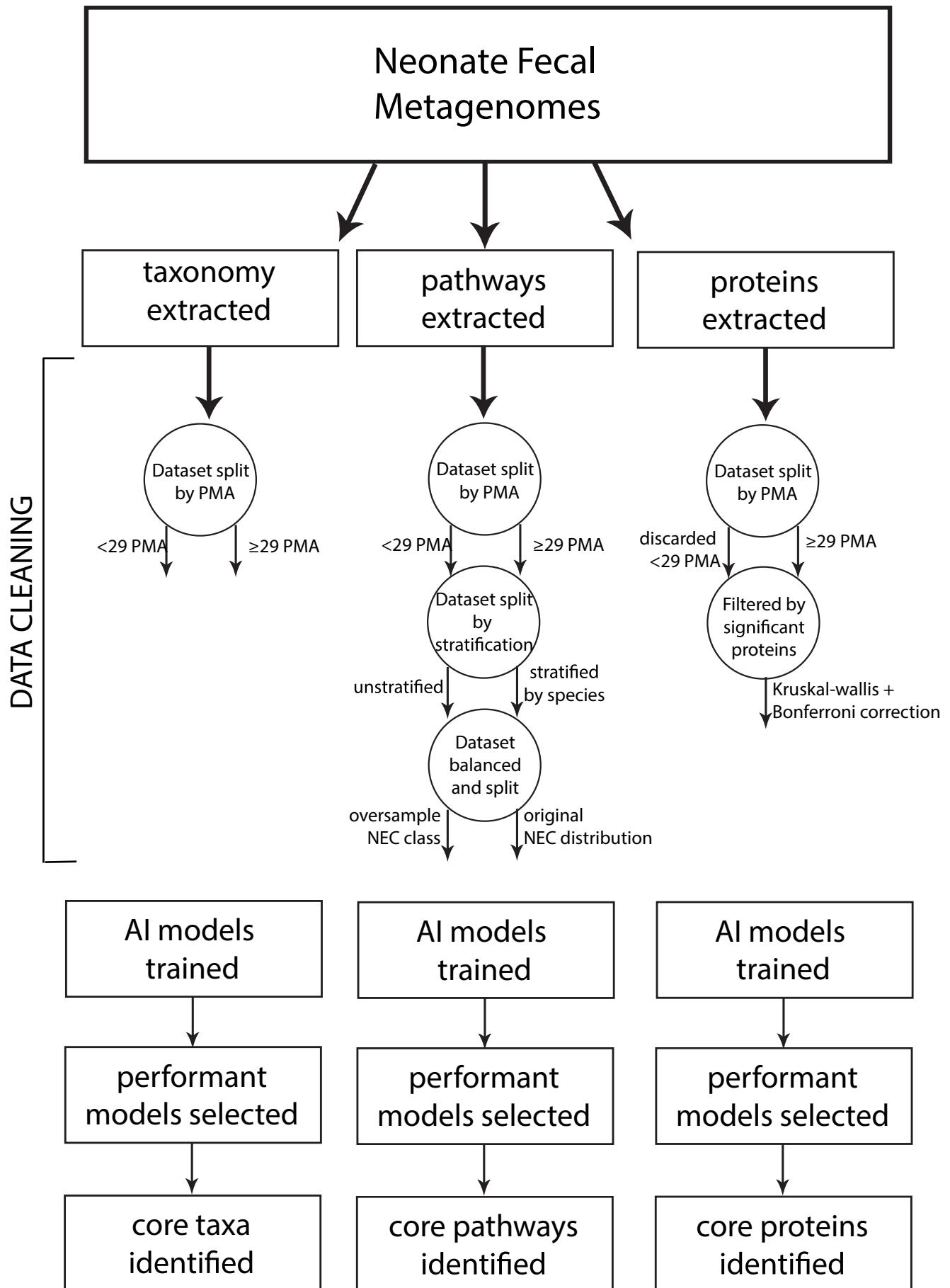

Supplement: Supplementary Figure 2 — Schematic of the metagenomic data modeling strategy. [file Data_Sheet_2.PDF]

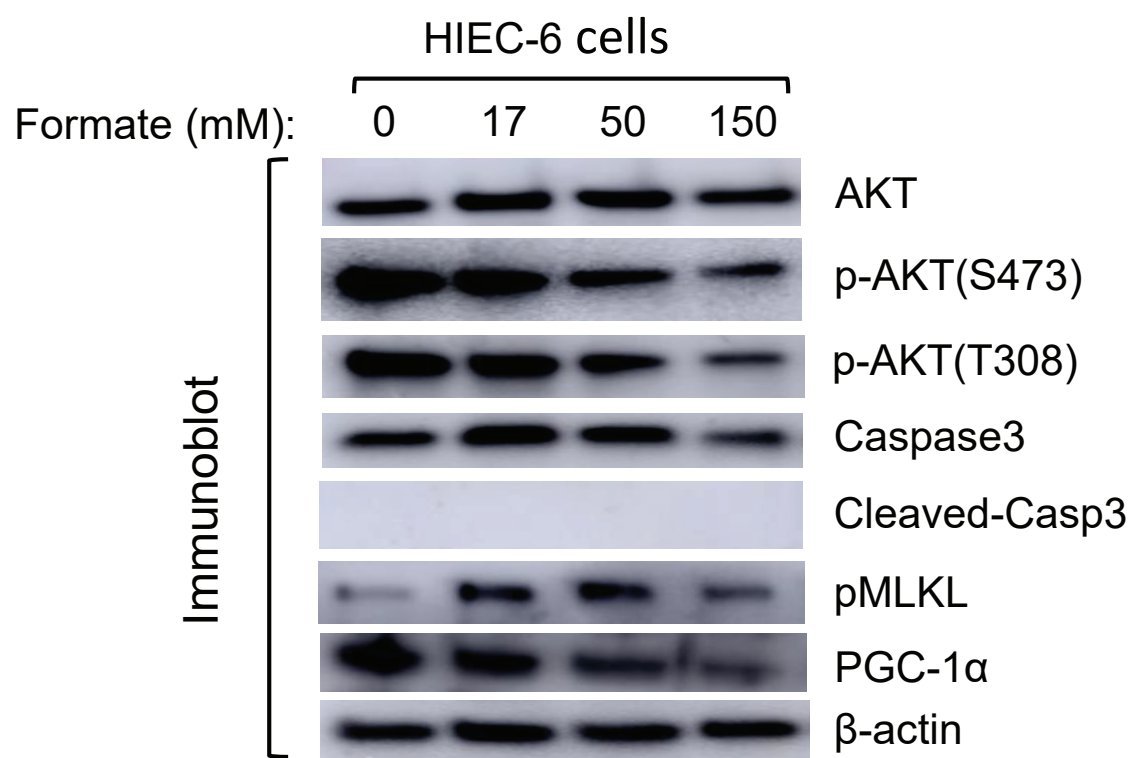

Supplement: Supplementary Figure 3 — Western blot showing relevant protein effector protein changes in dose response to formate by HIEC-6 cells (human intestinal epithelial cells 6). (mM = milli-molar), AKT = protein kinase B) [pMLKL = phospho-mixed lineage kinase domain-like protein (MLKL) (PGC1a = peroxisome proliferator-activated receptor gamma coactivator)-1alpha]. [file Data_Sheet_3.PDF]
