# Supplementary material for: Metabolic model of necrotizing enterocolitis in the premature newborn gut resulting from enteric dysbiosis
Source: Front Pediatr. 2022 Aug 23;10:893059. doi: 10.3389/fped.2022.893059 (PMC9445129; doi:10.3389/fped.2022.893059)
Supplement: Supplementary Table 2 — Histological grading scale for neonatal mouse model of NEC. [file Table_2.DOCX]

| **Pathological score** | **Pathological change** |
| --- | --- |
| **0** | Intestinal mucosa and villi are intact and  the structure was completely normal |
| **1** | Slight submucosal and/or lamina propria separation |
| **2** | Moderate submucosal and/ or lamina propria separation,  submucosal and/or muscular edema |
| **3** | Severe submucosal and/or lamina propria separation,  submucosal and/or muscular edema, and local villi shedding |
| **4** | Intestinal villi are small and accompanied by intestinal necrosis |

**Supplemental Table 2**. Histological grading scale for neonatal mouse model of NEC.
